# The ecology of scale: impact of volume on coalescence and function in methanogenic communities

**DOI:** 10.1098/rsfs.2022.0089

**Published:** 2023-06-09

**Authors:** Pawel Sierocinski, Peter Stilwell, Daniel Padfield, Florian Bayer, Angus Buckling

**Affiliations:** ESI, Biosciences, University of Exeter, Penryn Campus, Penryn TR10 9FE, UK

**Keywords:** microbial ecology, engineered ecosystems, community biology

## Abstract

Engineered ecosystems span multiple volume scales, from a nano-scale to thousands of cubic metres. Even the largest industrial systems are tested in pilot scale facilities. But does scale affect outcomes? Here we look at comparing different size laboratory anaerobic fermentors to see if and how the volume of the community affects the outcome of community coalescence (combining multiple communities) on community composition and function. Our results show that there is an effect of scale on biogas production. Furthermore, we see a link between community evenness and volume, with smaller scale communities having higher evenness. Despite those differences, the overall patterns of community coalescence are very similar at all scales, with coalescence leading to levels of biogas production comparable with that of the best-performing component community. The increase in biogas with increasing volume plateaus, suggesting there is a volume where productivity stays stable over large volumes. Our findings are reassuring for ecologists studying large ecosystems and industries operating pilot scale facilities, as they support the validity of pilot scale studies in this field.

## 1. Introduction

Natural microbial systems operate on spatial scales ranging from a single droplet to a global ocean [1]. Engineered systems also span orders of magnitude of volume, ranging from nano-scale microbial fuel cells to large catchment wastewater treatment plants. These larger engineered systems pose particular challenges for scientific research: replicated experimental manipulations are either impossible (not enough comparable facilities exist) or are simply too expensive and risky. It is therefore necessary to turn to much smaller and experimentally tractable systems and hope that results can at least be qualitatively scaled up [2].

Spatial scale can however be very important for community composition and function. For example, positive relationship between species diversity and area (or volume) is a well-established pattern in macrobes [3] and more recently in microbial communities [4–6]. There are a number of reasons for this pattern, including smaller areas having less habitat heterogeneity, less immigration and greater extinction rates [3,7]. Crucially, diversity is often positively associated with community functions, such as productivity and metabolism [8,9]. Furthermore, the relative importance of localized biotic interactions versus abiotic conditions increases with decreasing scale, meaning that the predictability of communities' structure and functions may decrease at smaller scales [10]. These differences mean that the conclusions reached by examining one scale may have little relevance to another.

However, it is possible to provide some insight into scalability by conducting experiments at different laboratory scales. Here, we employ such an approach for methane production by anaerobic digestion. Anaerobic digestion is increasingly carried out at industrial scales of thousands of cubic metres, and inferences about the process are frequently made from much smaller scale reactors. This may be problematic because methane-producing communities (MPCs) can show links between their biodiversity and function (specifically, methane production) [11,12]. Furthermore, both composition and methane production change with abiotic conditions, and the extent to which this is the case may decrease with scale.

We specifically focus on the importance of scale in the outcome of mixing microbial communities (community coalescence [13,14]) in three MPCs at three volumes (3 ml, 30 ml and 300 ml). Community coalescence has been characterized in microbial communities from anaerobic digesters [2], water [15], soil [16], gut [17] and synthetic communities [18–20]. In MPCs, community coalescence leads to the domination of the best individual community within the mix [2,21], a finding consistent with recent theory [14,22]. Crucially, the best-performing community also produces the most methane, meaning that community coalescence on average increases methane production. This is because a large part of community metabolism in MPCs is channelled into methane production by the methanogens [23], and the substrates that are not converted to methane accumulate as organic acids leading to drop in pH and dying off of the community. Increasing volume could potentially decrease the importance of coalescence because higher diversity may reduce variance in performance between communities because of increasing functional redundancy; but could also increase the importance if decreasing impact of localized interactions makes the outcome of coalescence more deterministic

## 2. Methods

### 2.1. Methane-producing communities and cultivation

We chose three communities based on their gas productivity in previous experiments: one high-, one intermediate- and one low-productivity community were selected. Six replicate mixed communities were generated by combining the three individual communities at equal ratios by volume, as previous experiments have shown that cell numbers were within the same order of magnitude for those communities [2]. We used three sizes of cultivation bottles: 10 ml, 100 ml and 1000 ml. Each cultivation bottle was inoculated with 3, 30 or 300 ml of the community mix or same volume of an individual community, yielding a total of six replicates of community mixes and four replicates of each individual community per volume tested. To each of the 3 ml, 30 ml and 300 ml communities, 200 µl, 2 ml and 20 ml of feed composed of 3.53 g l^−1^ casein, 1.17 g l^−1^ peptone, 1.17 g l^−1^ albumen, 47.07 g l^−1^ dextrin and 47.07 g l^−1^ sucrose (all compounds from Sigma) were added by syringe with a 100 mm needle, at the beginning of the experiment, and then each week for seven weeks immediately after gas measurements were taken. Reactors were incubated at 37°C, shaking at 180 rpm to keep them homogenized.

### 2.2. Gas measurements

Reactors were allowed to equilibrate to room temperature for 4 h prior to taking pressure measurements. Pressure measurements were taken each week using a digital manometer (Keller), after which all communities were fed and vessels re-pressured to 1100 mbar. Gas production was calculated by dividing the final pressure by the initial pressure and multiplying by the headspace volume, considering the reduction in headspace according to the feed volume. The headspace volumes were estimated for each of the bottle sizes prior to the experiment by taking the average of three measures of the weight of excess water required to completely fill a vessel after an inoculum of the ascribed size had first been added.

### 2.3. DNA extraction, amplicon construction and sequencing

Samples for DNA analyses were taken from each of the single communities and the mix at the beginning of the experiment, and from all replicates in all treatments at the end of the experiment. DNA was extracted from 1 ml of each sample following the QIAmp DNA Stool Mini Kit (QIAGEN) with bead beating parameter set to 3000 rpm for 45 s. 16S rRNA gene libraries were constructed using primers designed to amplify the V4 region and multiplexed [24]. Amplicons were generated using a high-fidelity polymerase (Kapa 2G Robust) and purified using the Agencourt AMPure XP PCR purification system and quantified using a fluorometer (QuBit, Life Technologies). The purified amplicons were then pooled in equimolar concentrations by hand based on QuBit quantification. The resulting amplicon library pool was diluted to 2 nM with sodium hydroxide and 5 µl transferred into 995 µl of HT1 (Illumina) to give a final concentration of 10 pM. Six hundred microlitres of the diluted library pool was spiked with 10% PhiX Control v3 and placed on ice before loading into Illumina MiSeq cartridge following the manufacturer's instructions. The sequencing chemistry used was MiSeq Reagent Kit v2 (500 cycles) with ren metrics of 250 cycles for each paired-end read using MiSeq Control Software 2.2.0 and RTA 1.17.28.

### 2.4. Analysis of gas production

Cumulative gas production per gram of inoculum was analysed using a two-way ANOVA, with the *post hoc* Tukey HSD test (R, aov and Tukey HSD functions), looking for the effects of the volume in which the communities were incubated, as well as the community origin (including the mixed communities) and the interaction between volume and community origin. To account for any effects of the initial condition of the different communities, the first week of gas production was removed from analyses. All analyses were conducted in R.

### 2.5. Analysis of sequenced samples

MiSeq amplicon reads were merged using Illumina-utils software [25]. We quality-filtered only the mismatches in the overlapping region between read pairs using a minimum overlap (-min-overlap-size) of 200 nt and a minimum quality Phred score (-min-qual-score) of Q20. We allowed no more than five mismatches per 100 nt (−P 0.05) over the 200 nt overlapping region.

Reads that fulfilled the quality criteria were analysed using the ‘dada2’ and ‘phyloseq’ [26,27] packages in R (v 3.5.0). The standard full-stack workflow was followed; error rates were estimated, inferred and merged sequences, constructed a sequence table, removed chimeric sequences and assigned taxonomy. Forward and reverse reads were truncated between 25 and 250 nucleotide positions due to poor quality scores. Assembled amplicon sequence variants (ASVs) were assigned taxonomy using the Ribosomal Database Project [28]. FastTree was used to estimate the phylogenetic tree using an approximate maximum-likelihood method [29]. Before further analyses were undertaken, reads that had not been assigned taxonomy at the phylum level were filtered out, as were any ASVs that were present in less than 5% of all samples, as well as reads assigned as either cyanobacteria or chloroplasts. Post-processing and filtering, all 54 samples remained for downstream analyses. The maximum number of reads in a single sample was 79 073, the minimum 44 393, with a mean of 58 623. For alpha diversity analysis, samples were rarefied to even depth of 44 000 using GUniFrac package in R [30].

To compare communities, we first generated a Bray–Curtis community distance matrix using the R package ‘phyloseq’. The dissimilarity was visualized using NMDS, calculated using metaMDS command in R. To ascertain whether one of the mixed-volume communities was closer in composition to one of the origin communities at the end of the experiment, we performed a general linear mixed model (restricted maximal likelihood) design, with the Bray–Curtis distance from each of the individual communities to each mixed-community volume nested within each mixed-community replicate. We used adonis function in vegan package to run the PERMANOVA analysis on the Bray–Curtis dissimilarity. The alpha diversity measures, Chao1 and Pielou evenness were calculated using vegan package in R [31]. The data are available in European Nucleotide Archive under the id PRJEB61013.

## 3. Results

### 3.1. Gas production

Data from three out of four large volume (300 ml inoculum) community 31 treatments were lost due to pressure from the gas generated blowing the tops off the bioreactors. To account for this, two separate analyses were carried, one with all data from community 31 removed, and one with all data from large volume treatments removed.

**Figure 1.**
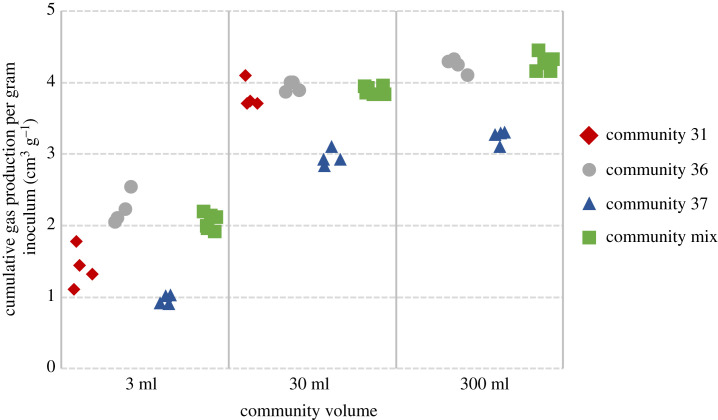
Cumulative gas production per gram of original inoculum by treatment (community 31—red; community 36—grey; community 37—blue; community mix—green).

For the analysis that excluded community 31, there was no significant interaction between volume and community on the cumulative amount of gas produced per gram of inoculum (figure 1; ANOVA, *F*_4,33_ = 2.273, *p* = 0.082). However, gas production differed between communities (ANOVA, *F*_2,37_ = 9.373, *p* < 0.0001): the mixed community and community 36 produced more gas per gram of inoculum than community 37 (*post hoc* Tukey pairwise comparisons: *p* < 0.050) but did not differ from each other. Gas production per gram of inoculum differed between volumes (ANOVA, *F*_2,37_ = , *p* < 0.0001), with greater volumes resulting in more gas (*p* < 0.05 for all pairwise comparisons).

Qualitatively similar conclusions were reached when data from all the 300 ml bioreactors were excluded. There was, however, a significant interaction between community and volume (ANOVA *F*_3,28_ = 8.33, *p* < 0.001). Specifically, *post hoc* Tukey HSD tests showed that medium volume (30 ml) inocula mixed communities and those of communities 31 and 36 did not differ significantly in gas production, but produced more gas than community 31 (*p* < 0.05, in all cases). In smaller volume (3 ml) inocula, the mixed communities and community 36 produced a greater amount of gas per gram of inoculum than community 31 (*p* < 0.05, in both cases), which produced a greater amount of gas per gram of inoculum than community 37 (*p* < 0.05) (figure 1). Overall, community origin had a significant impact on the cumulative amount of gas produced per gram of inoculum (ANOVA, *F*_3,28_ = 95.695, *p* < 0.001), and medium volumes resulted in more gas per gram of inoculum than small volumes (ANOVA, *F*_1,28_ = 1545, *p* < 0.001).

### 3.2. Community composition

#### 3.2.1. Within-community diversity

**Figure 2.**
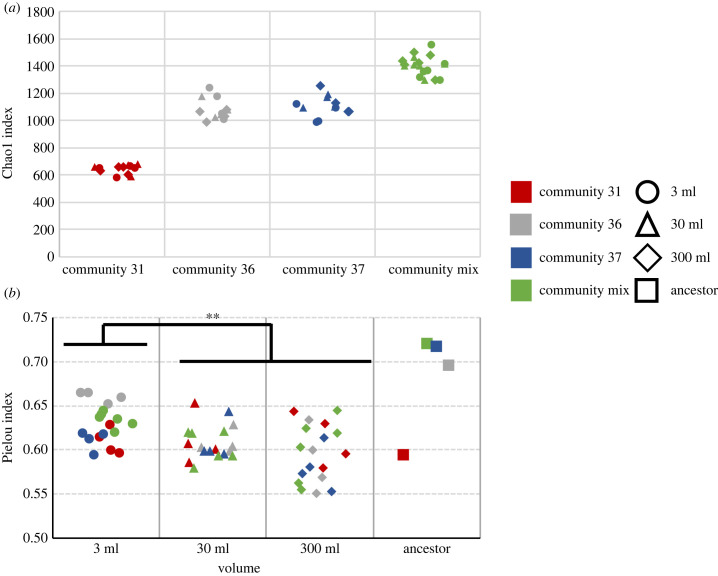
Chao1 estimate of the number of ASVs by treatment at endpoint (*a*); evenness of the samples by volume at endpoint and at the starting point (*b*). In both panels: community 31: red; community 36: grey; community 37: blue; mix: green; 3 ml volumes: circle; 30 ml volumes: triangle; 300 ml volumes: diamond; square: ancestor. Samples were rarefied to common depth of 44 000 reads prior to analysis.

The sequencing data revealed differences in alpha diversity between communities. There were significant differences in richness, as measured by the Chao1 index [32] between the communities (ANOVA, *F*_3,50_ = 306.9, *p* < 0.001) and Tukey *post hoc* analysis showed that the differences were significant between every pair of communities except for 36 and 37 (figure 2*a*, with mix > community 37 = community 36 > community 31; all differences *p* < 0.001 except for community 36 versus 37 where *p* = 0.8). There was no difference in richness between volumes. Evenness, measured by Pielou index, did not differ significantly between communities, but there was a difference between volumes (Kruskal–Wallis, *H*_2_ = 10.8, *p* = 0.0043). Pairwise comparisons showed that the 3 ml communities had greater evenness than both the 30 ml community and 300 ml community (*p* = 0.01 in both cases) but there was no significant difference between 30 ml and 300 ml communities (*p* = 0.28; see figure 2*b*).

#### 3.2.2. Between-community diversity

**Figure 3.**
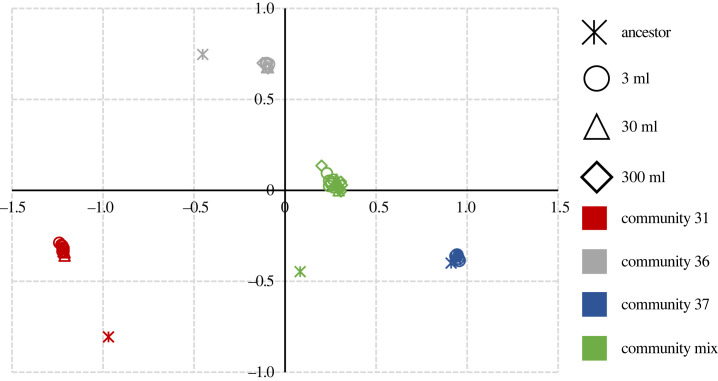
NMDS plot of Bray–Curtis for each community endpoint and ancestral community (stress = 0.057). Community 31: red; community 36: grey; community 37: blue; mix: green; 3 ml volumes: circle; 30 ml volumes: triangle; 300 ml volumes: diamond; ancestor: star.

All communities showed large shifts in their composition from their ancestral state, measured directly before inoculation, by the end of the experiment except community 37. The three final time point communities and the mix of all three were significantly different from each other based on the Bray–Curtis dissimilarity (figure 3; adonis, *p* < 0.001). Community 36, compared with communities 31 and 37, showed the greatest convergence with the mixed community from their ancestral states, suggesting taxa from community 36 were dominating the mixture. This is supported by the fact that 387 ASVs from community 36 were no more than twofold different from mixed communities, while in the case of communities 31 and 37 it was 146 and 319, respectively. Community 36 also had the most unique ASVs (5) compared to 4 and 0 for communities 31 and 37, respectively. There was also a small, but significant, impact of the volume treatment on between-culture diversity for each community type (adonis, *R*^2^ for communities 31, 36, 37 and mix, respectively, 0.23, 0.21, 0.22, 0.29; see electronic supplementary material, table, for details) but this was dwarfed by the differences between communities.

## 4. Discussion

The results of our experiment were broadly consistent with previous coalescence experiments in MPCs [2,21]. Mixing communities increases gas production to the level of the best-performing component community (community 36). Furthermore, community 36 also appeared to dominate the mixture. Crucially, the beneficial effect of community mixing operates in a qualitatively similar way across two orders of magnitude of volume, increasing the likelihood that the beneficial effect will scale up to industrial anaerobic digesters. While coalescence was largely scale independent, the volume of the reactors had a significant impact on the gas production of the communities: the larger the volume, the higher the productivity per gram of the inoculum. This increase was most pronounced from 3 ml to 30 ml, suggesting that at some point the further increases in the volume will have minimal impact on gas production. The 3 ml communities were most likely more prone to stochastic dilution effects meaning that some functions within communities were lost, which is consistent with our previous work on dilution to extinction, showing the importance of rare species and their impact on community function [12].

While there was no link between volume and species richness, communities from the 3 ml treatment were significantly more even than the communities from the 30 and 300 ml treatments. Similar negative link between area of a habitat and evenness has been found in bird populations [33] and in pollinating insects [34], where it is speculated that this is caused by small habitats not being capable of feeding specialist species. In our communities, this might also be the case that some substrates exist in the smaller volume communities at such low levels that no single species can specialize to live on them consistently. This may then have opened up resources for generalist oligotrophic scavenger species and increases the evenness of the ecosystem.

The Chao1 estimate of the numbers of ASVs (richness) was greater in the mixed community than any of the individual communities. Richness did not, however, correlate with gas production. Previous correlational and experimental work has shown a link between diversity and productivity within a single community [12], and the lack of relationship in this experiment is most likely caused by the small number of communities used resulting in other community differences obscuring any effects of richness.

Our experiment shows that scale of the experiment does impact productivity of the community and has some impact on its evenness, but those differences do not obscure the ecological process we wanted to examine. This makes us optimistic that small-scale ecological experiments do shed a light on what is going on much larger scales. The flattening of the productivity curve over different orders of magnitude suggests that there is a volume where productivity stagnates and experiments done on that scale will be equivalent to the industrial scale. Our findings are reassuring to those who study ecology of whole ecosystems in laboratory conditions but also to industries operating on industrial scales that worry about validity of pilot scale experiments to their much larger operational systems.

## Data Availability

Sequencing data are available in the European Nucleotide Archive under the id PRJEB61013. Supplementary material is available online [35].
